# Toxic Megacolon: A Sequelae of Clostridioides difficile Infection in a Case of Necrotizing Fasciitis

**DOI:** 10.7759/cureus.53034

**Published:** 2024-01-27

**Authors:** Abhilasha Bhargava, Chandrashekhar Mahakalkar, Shivani Kshirsagar, Akanksha Yachmaneni

**Affiliations:** 1 General Surgery, Jawaharlal Nehru Medical College, Datta Meghe Institute of Higher Education & Research, Wardha, IND

**Keywords:** necrotizing fasciitis, antibiotic-associated diarrhea, toxic megalcolon, clostridium difficile infections, colitis

## Abstract

Necrotizing fasciitis is an illness that ascends quickly and affects the fascia, subcutaneous tissues, and deeper skin layers. To combat this infection, strong antibiotics are used along with prompt debridement. Frequent usage of such drugs is connected to antibiotic-associated diarrhea and colonic illnesses like colitis. High-spectrum antibiotic usage over an extended period of time can alter the gut microbiota, which promotes the growth of commensal bacteria including *Staphylococcus aureus* and *Clostridioides* *difficile* (previously known as *Clostridium difficile)* resulting in complications such as toxic megacolon.* C. difficile* infection can result in extreme inflammation and colon dilatation leading to toxic megacolon. In order to effectively treat necrotizing fasciitis, a timely diagnosis and vigorous management are essential; failing of which may have fatal consequences such as sepsis and even mortality. We present a case of a 56-year-old male, suffering from necrotizing fasciitis of the left lower limb which further complicated to toxic megacolon and caused mortality of the patient. Timely presentation and early diagnosis can be helpful in better prognosis, which in the context of this case was delayed; had the patient presented to the hospital earlier, there were chances of preventing mortality.

## Introduction

Necrotizing fasciitis is a potentially life-threatening infection that affects deeper skin tissues. This is a rapidly spreading infection that affects the deeper layers of skin, subcutaneous tissues, and fascia. Compromised immune systems can cause more susceptibility to necrotizing fasciitis. It is typically caused by different bacteria, often including certain strains of *Streptococcus* or *Staphylococcus*. *Clostridioides difficile* (*C. difficile*) is a gram-positive bacilli anaerobic microorganism listed as one of the major reasons of antibiotic-associated diarrhea and other colonic infections such as colitis, which can cause toxic megacolon leading to adversity. Prolonged use of high-spectrum antibiotics can disrupt the normal microbial flora of the intestine leading to the growth of commensal microorganisms such as *C. difficile* and *Staphylococcus aureus* [1]. *C. difficile* has been associated with clindamycin-associated colitis and is surfacing as a global threat due to its severe clinical presentations in humans. Most of the infections caused by *C. difficile* affect the colon, but extra-intestinal infections have also been reported [2]. *C. difficile* is classified into two variants, toxicogenic and non-toxicogenic with a commensal microbial status [3,4]. *C. difficile* infection can lead to toxic megacolon, which involves severe inflammation and dilatation in the colon. The connection lies in how treatment of necrotizing fasciitis with antibiotics can cause compromised immune systems and lead to severe infections like *C. difficile *infection.

## Case presentation

**Figure 1:**
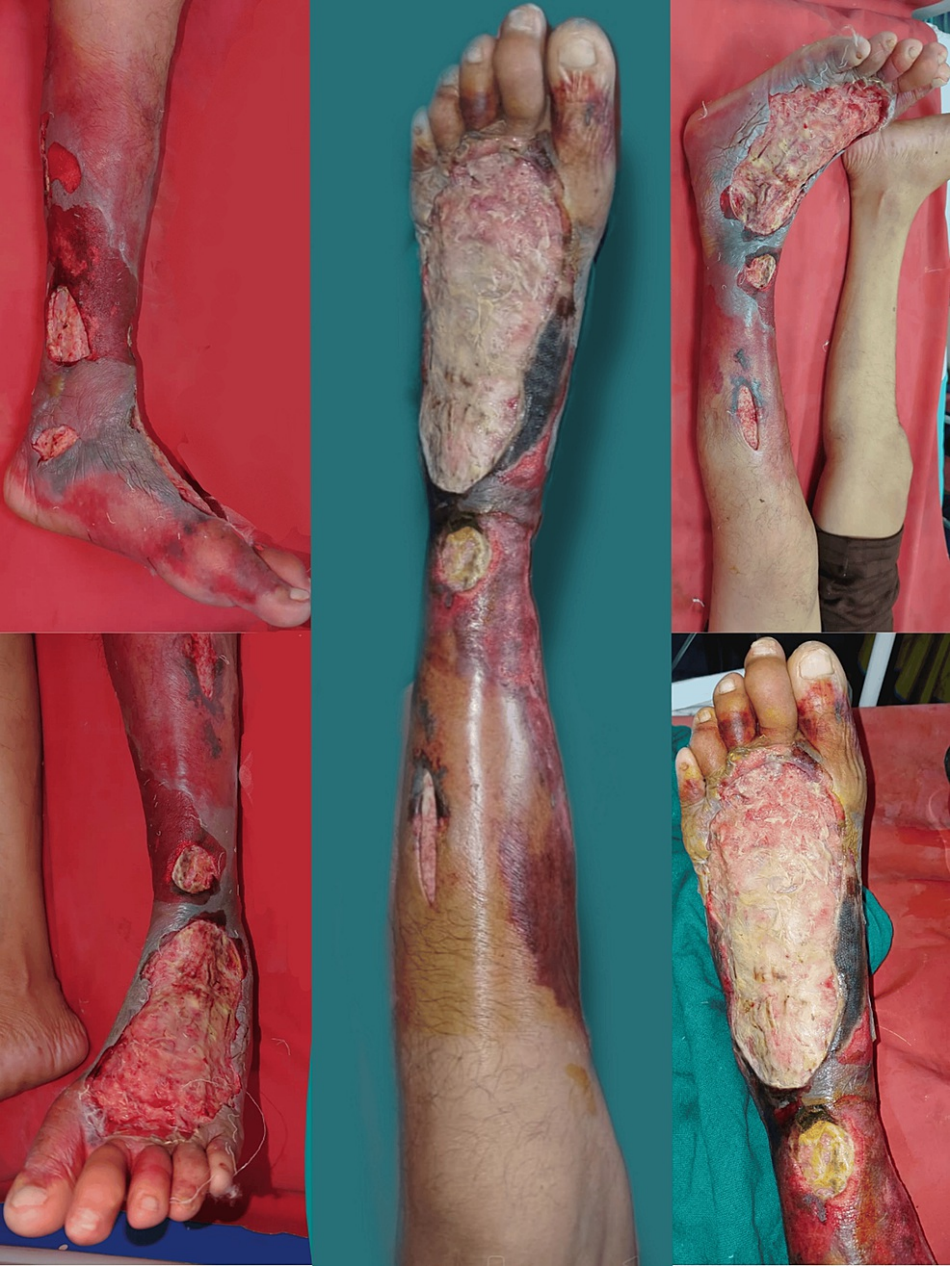
Clinical presentation of a necrotizing fasciitis of the left lower limb on admission

A 56-year-old male came to the hospital with complaints of pain with swelling of the left lower limb for 20 days with an ulcer over the dorsum of the left lower limb for 15 days. The patient had a known case of diabetes mellitus and initially presented with cellulitic changes over the left lower limb to the knee which eventually gave way to an ulcer formation over the foot. The ulcer was insidious in onset and gradually progressed to its current size of 8 x 7 cm (Figure 1).

**Figure 2:**
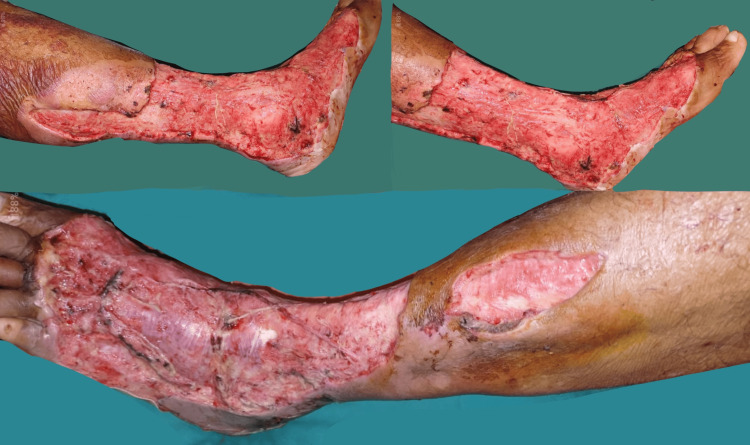
Clinical image showing the status of the left lower limb after the first debridement

On general examination, the patient was febrile, normotensive, tachycardic, and tachypneic. On admission, his leukocytic count was 15,000 cumm, pertaining to sepsis. As per protocol of necrotizing fasciitis management, the patient was started on higher antibiotics such as meropenem injection 500 mg IV BD, metronidazole injection 500 mg TDS, and clindamycin injection 600 mg IV BD. He was taken for emergency debridement of wound over the left lower limb with subsequent release incisions over cellulitic regions to save the rest of the limb (Figure 2).

**Figure 3:**
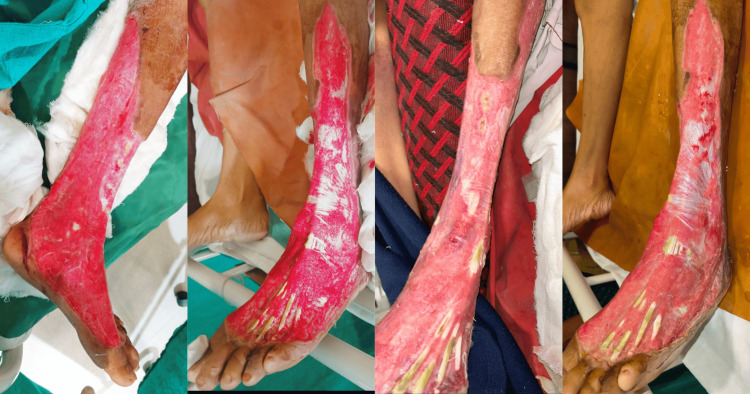
Clinical image showing the presence of healthy granulation tissue post sequential debridements There is evidence of minimal slough with exposed tendons.

The patient was subjected to strict ICU monitoring with daily magnesium sulfate dressing of the left lower limb along with limb elevation. After two subsequent wound debridements, his wound was healthy with granulation tissue with minimal slough (Figure 3).

**Figure 4:**
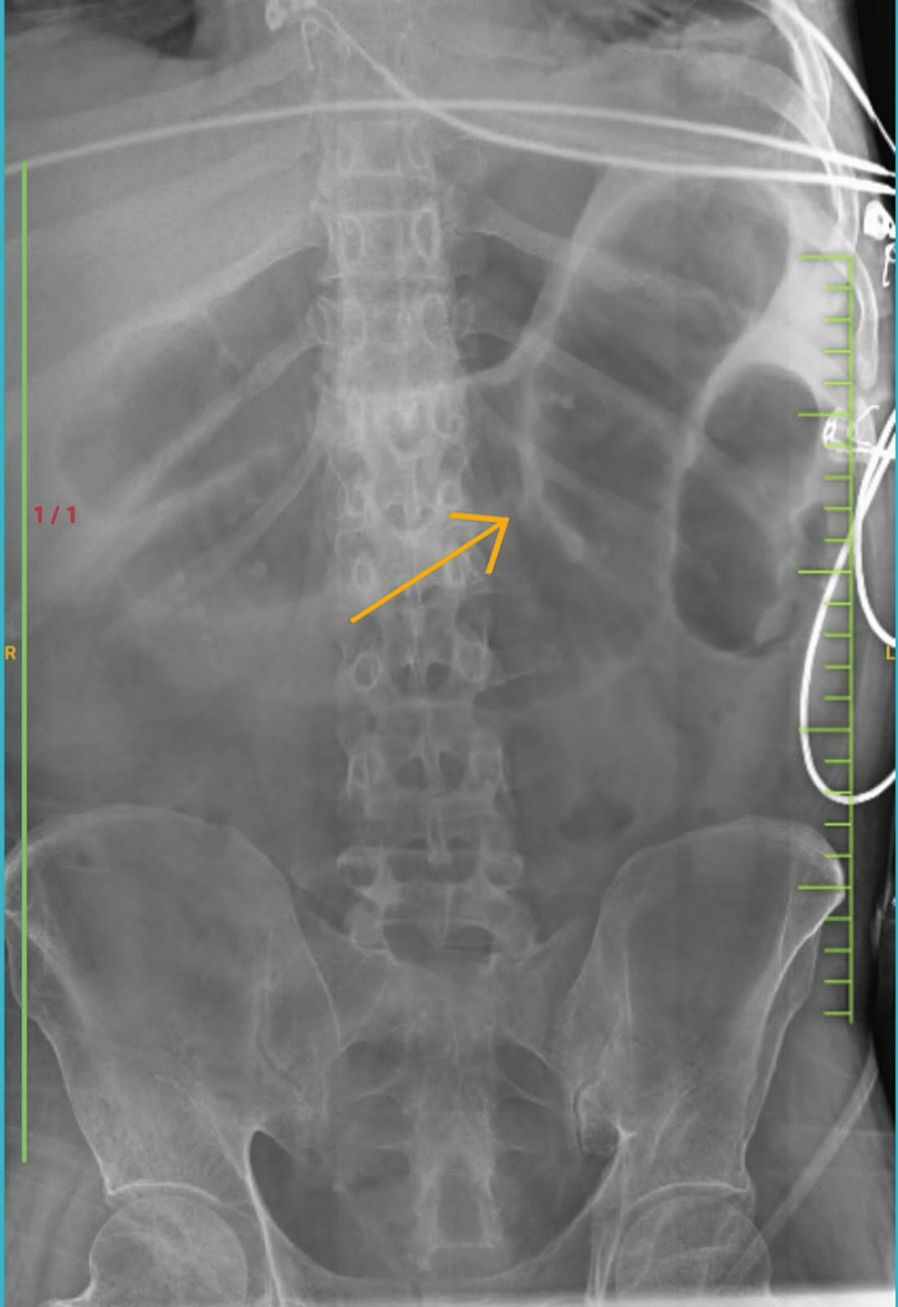
Abdominal radiograph showing a dilated bowel segment suggestive of toxic megacolon, a sequelae of pseudomembranous colitis due to Clostridium infection The arrow indicates a dilated bowel segment with a 6.2 cm diameter.

Postoperatively as per wound swab culture sensitivity test, he was started on tab linezolid 600 mg PO BD. On postoperative day 8, the patient started complaining of pain in abdomen, loss of appetite with distension, and multiple episodes of passing liquid consistency stools. He was started on Lactobacillus supplements, but his condition did not improve. His Ryle’s tube collection was of 500-700 ml bilious everyday. A stool test by chemiluminescence immunoassay was sent for the patient, which was positive for toxigenic *C. difficile* glutamate dehydrogenase (GDH) with toxin A/B positive. The patient was diagnosed as pseudomembranous colitis and was thereby started on tab vancomycin 250 mg QID. As per blood reports, his counts were in the increasing trend along with deranged liver and kidney function test results, which were not a promising sign. The patient was gradually inclining toward multiple organ failure with deranged liver and kidney function tests. As per blood culture sensitivity report, the patient was started on colistin injection 150 mg BD. On erect abdomen radiograph, we could appreciate the distended bowel loops giving an impression of toxic megacolon (Figure 4).

**Figure 5:**
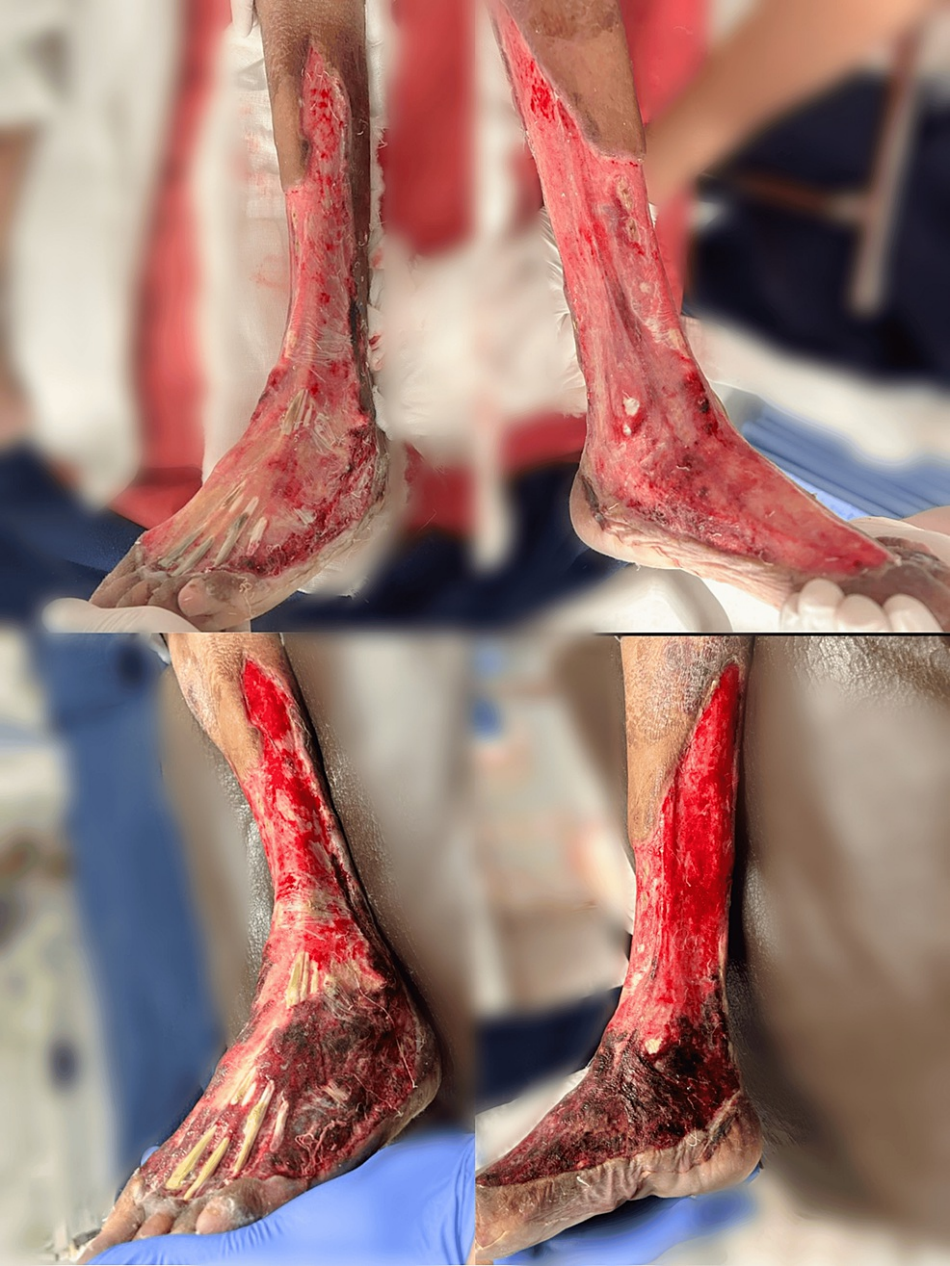
Clinical images showing the worsening of the wound due to terminal hypoxia after the patient's condition deteriorated

The patient deteriorated into septicemia again, having terminal hypoxia which worsened the healthy wound over the left limb post debridement (Figure 5).

All these factors culminated to cardiorespiratory arrest secondary to sepsis with septic shock and lead to mortality of the patient.

## Discussion

Necrotizing fasciitis, being a severe inflammation, has been recommended to have timely diagnosis and prompt intervention; failing of which might lead to adverse outcomes and even mortality [5,6]. Higher doses of advanced antibiotics are recommended for the management of necrotizing fasciitis to cater to the septic focus. These antibiotics may lead to diarrhea due to disruption of the gut microbiota leading to subsequent disorders as colitis which when mismanaged or not diagnosed on time results in a toxic megacolon [7]. The main causal microorganism behind these complications is *C. difficile*. Most of the cases of antibiotic-associated diarrhea, colitis, and toxic megacolon are caused by uncontrolled growth of *C. difficile* and are reported to cause toxic megacolon, intestinal perforation, multi-organ failure, and even death [8]. Underlying etiologies for toxic megacolon are ulcerative colitis and Crohn’s disease with a continuously increasing incidence. *C. difficile* was reported as a 4.3% incidence of toxic megacolon, and mortality rates are variably noted [9]. Management of toxic megacolon is done by a multiparametric team of experts with diagnostic methods which do not require prior bowel preparations such as sigmoidoscopy or proctoscopy in lieu of complete colonoscopy attributed to the risk of intestinal perforation. Intestinal perforation has been linked to mortality in cases of toxic megacolon [10]. Research studies indicate discontinuation of empirical antibiotics or high-dose medications such as steroids in cases with complications of antibiotic-associated diarrhea or those developing complexities such as toxic megacolon [10,11].

## Conclusions

Timely diagnosis and prompt intervention are crucial for managing necrotizing fasciitis, as failure can lead to adverse outcomes and even death. Management of this patient involved a multiparametric team of surgeons, a gastroenterologist, and a critical care team.

## References

[1] Johanesen PA, Mackin KE, Hutton ML, Awad MM, Larcombe S, Amy JM, Lyras D (2015). Disruption of the gut microbiome: Clostridium difficile infection and the threat of antibiotic resistance. Genes (Basel).

[2] Elliott B, Androga GO, Knight DR, Riley TV (2017). Clostridium difficile infection: evolution, phylogeny and molecular epidemiology. Infect Genet Evol.

[3] Vedantam G, Clark A, Chu M, McQuade R, Mallozzi M, Viswanathan VK (2012). Clostridium difficile infection: toxins and non-toxin virulence factors, and their contributions to disease establishment and host response. Gut Microbes.

[4] Burke KE, Lamont JT (2014). Clostridium difficile infection: a worldwide disease. Gut Liver.

[5] Sayedy L, Kothari D, Richards RJ (2010). Toxic megacolon associated Clostridium difficile colitis. World J Gastrointest Endosc.

[6] Morgan MS (2010). Diagnosis and management of necrotising fasciitis: a multiparametric approach. J Hosp Infect.

[7] Högenauer C, Hammer HF, Krejs GJ, Reisinger EC (1998). Mechanisms and management of antibiotic-associated diarrhea. Clin Infect Dis.

[8] Mullish BH, Williams HR (2018). Clostridium difficile infection and antibiotic-associated diarrhoea. Clin Med (Lond).

[9] Desai J, Elnaggar M, Hanfy AA, Doshi R (2020). Toxic megacolon: background, pathophysiology, management challenges and solutions. Clin Exp Gastroenterol.

[10] Strich JR, Heil EL, Masur H (2020). Considerations for empiric antimicrobial therapy in sepsis and septic shock in an era of antimicrobial resistance. J Infect Dis.

[11] Liggett MR, Alam HB (2023). Management of severe colitis and toxic megacolon. Clin Colon Rectal Surg.

